# Association of Low Muscle Mass With Cognitive Function During a 3-Year Follow-up Among Adults Aged 65 to 86 Years in the Canadian Longitudinal Study on Aging

**DOI:** 10.1001/jamanetworkopen.2022.19926

**Published:** 2022-07-01

**Authors:** Anne-Julie Tessier, Simon S. Wing, Elham Rahme, José A. Morais, Stéphanie Chevalier

**Affiliations:** 1 School of Human Nutrition, McGill University, Sainte-Anne-de-Bellevue, Quebec, Canada; 2 Research Institute of the McGill University Health Centre, Montreal, Quebec, Canada; 3 Department of Medicine, McGill University, Montreal, Quebec, Canada

## Abstract

**Importance:**

Cross-sectional studies have shown that combined low muscle mass and strength are associated with cognitive impairment. Whether low muscle mass, reflective of physiologic reserve, is independently associated with faster cognitive decline remains unknown.

**Objective:**

To investigate the associations between low muscle mass and cognitive decline in 3 distinct domains among adults aged at least 65 years.

**Design, Setting, and Participants:**

The Canadian Longitudinal Study on Aging is a prospective population-based cohort study of community-dwelling adults. Enrollment occurred from 2011 to 2015 with a 3-year follow-up. Analyses for this study were conducted on those aged at least 65 years from April 24 to August 12, 2020.

**Exposure:**

Appendicular lean soft tissue mass (ALM) was assessed by dual energy x-ray absorptiometry. Low ALM was identified using the sex-specific Canadian cut points.

**Main Outcomes and Measures:**

Memory was assessed using the Rey auditory verbal learning test. Executive function was assessed using the mental alternation test, Stroop high interference (words/dot) test, the animal fluency test, and the controlled oral word association test. Psychomotor speed was assessed using computer-administered choice reaction time. Composite scores by domain were created.

**Results:**

Of 8279 participants, 4003 (48%) were female, 8005 (97%) were White, and the mean (SD) age was 72.9 (5.6) years. A total of 1605 participants (19.4%) had low ALM at baseline. Participants with low ALM were older, had lower body mass index and physical activity level. The presence of low ALM at baseline was associated with faster 3-year cognitive decline in executive functions and psychomotor speed from multiple linear regressions. After adjusting for covariates including age, level of education, percentage body fat, and handgrip strength, low ALM remained independently associated with executive function decline (standardized β: −0.032; *P* = .03) only. Low ALM was not associated with memory.

**Conclusions and Relevance:**

This cohort study found longitudinal associations between low ALM and cognition in aging. Identification of older adults with low muscle mass, a targetable modifiable factor, may help estimate those at risk for accelerated executive function decline. Further longer-term investigation of associations is warranted.

## Key Points

**Question:**

Is low muscle mass associated with declines in different cognitive domains over 3 years?

**Findings:**

In cohort study that included 8279 older adults, the presence of low muscle mass was significantly and independently associated with faster subsequent executive function decline over 3 years.

**Meaning:**

These findings suggest the potential for clinical screening of older adults to identify those with low muscle mass to assist in risk detection of cognitive impairment development.

## Teaser

This cohort study investigates the associations between low muscle mass and cognitive decline in the domains of memory, executive functions, and psychomotor speed among adults aged at least 65 years.

## Introduction

Dementia is increasingly prevalent with age and negatively affects the quality of life of both patients and families.^1^ Unfortunately, the pathological changes responsible for dementia appear to be irreversible by the time of diagnosis. Treatments are few, very limited in efficacy, and target symptoms.^2^ Therefore, identifying modifiable biomarkers that may estimate the risk for subsequent cognitive decline is critical. Such measurable biomarkers could identify high-risk patients appropriate for further testing of potential disease-modifying therapies.

Depending on definitions, sarcopenia prevalence ranges from 10% to 40% in community-dwelling older adults.^3,4^ Sarcopenia, originally characterized by age-related low skeletal muscle mass also includes low muscle strength and physical performance,^5^ but a consensus definition has not been reached.^6,7,8^ The pathogenetic mechanisms proposed for accelerated cognitive decline are similarly implicated in sarcopenia—lack of anabolic hormones, vascular diseases, chronic inflammation, insulin resistance, neuronal dysfunction—suggesting both may be linked.^9^ Sarcopenia may be prodromal to the onset of cognitive impairment^10^ and may represent a sensitive marker of cognitive decline. However, the sarcopenia construct, including 3 related but distinct components, precludes identifying independent associations of each with cognition, which could be important to guide future mechanistic investigations. Physical and cognitive functions are dually related, and as such, are part of the frailty^11^ and the motoric cognitive risk syndromes.^12^ Lower handgrip strength has recently been associated with higher incident dementia risk and mortality in the UK Biobank.^13^ However, little attention has been brought to muscle mass. Beyond its role in body strength and function, muscle is an endocrine organ releasing several myokines involved in brain functions.^14,15^ To date, few cross-sectional studies have explored the relationship between muscle mass or the combination of low muscle mass and strength with cognitive impairment. Conclusions were not uniform with some showing an association between the 2 conditions^16,17,18^ and others not.^19,20,21,22^ To our knowledge, no studies have explored the relationship between muscle mass, independently of strength, and subsequent cognitive decline. To address this gap, we examined the associations between low appendicular lean soft tissue mass (ALM, proxy for skeletal muscle mass) and 3-year decline in 3 cognitive domains—memory, executive functions, and psychomotor speed—in free-living older adults of the Canadian Longitudinal Study on Aging. Given the multiple possible links between muscle and cognition, we hypothesized that low muscle mass would be associated with decline in all 3 cognitive domains studied.

## Methods

### Study Population

The design and methods of the nationally representative Canadian Longitudinal Study on Aging (CLSA) have been described.^23^ Between 2011 and 2015, the comprehensive cohort enrolled 30 097 free-living male and female participants aged 45 to 85 years across 11 cities in 7 provinces. Participants were able to speak French or English, were free of cognitive impairment that precluded the ability to provide informed consent at time of recruitment and underwent in-depth neuropsychological, body composition, physical function, and clinical assessments at baseline and at 3-year follow-up. Assessments are repeated every 3 years for 20 years.

**Figure 1.**
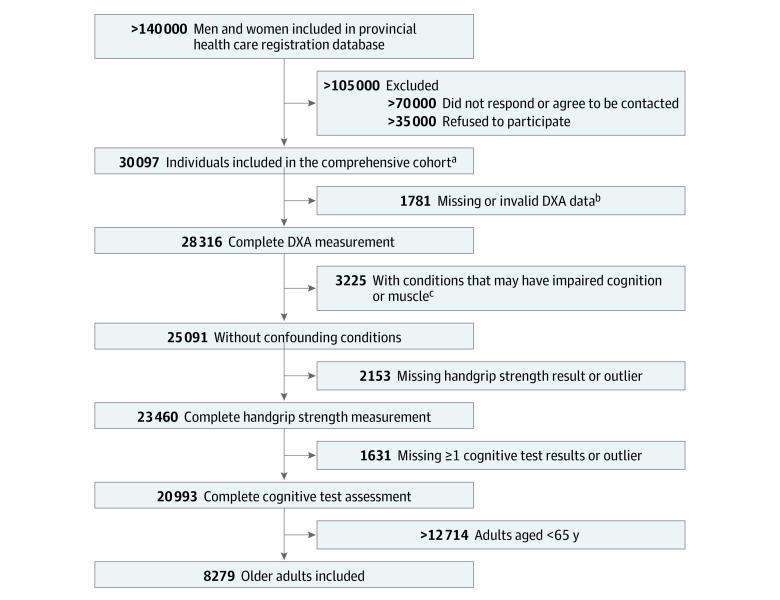
Diagram of Participants in the Canadian Longitudinal Study on Aging Cohort ^a^Exclusion criteria are described by Raina et al,^23^ 2009. ^b^Determined using Bland-Altman agreement plot that compared participants’ weight measured by DXA and by scale as described by Tessier et al,^3^ 2019. ^c^These conditions included multiple sclerosis, Alzheimer disease, effects from stroke or transient ischemic attack, Parkinson disease, surgery within last 3 months, polio, chemotherapy within last 4 weeks, traumatic brain injury with memory problem, positive screen for posttraumatic stress disorder, receiving dialysis treatment.

The subsample used in the current analyses included participants aged at least 65 years, with complete baseline cognitive, body composition, and handgrip strength assessments. Participants with a clinical condition possibly affecting the exposure or outcome were excluded, namely those with multiple sclerosis, Alzheimer disease, sequelae of stroke or transient ischemic attack, Parkinson disease, surgery within the past 3 months, polio, chemotherapy within the past 4 weeks, traumatic brain injury with memory problem, positive screen for posttraumatic stress disorder, or receiving dialysis treatment. Participants with inaccurate dual-energy x-ray absorptiometry (DXA) measurement were excluded.^3^ Our study sample included 8279 participants (4276 male participants and 4003 female participants) (Figure 1). Analyses were conducted from April 24 to August 12, 2020. The CLSA study was approved by the research site ethics boards and all participants of the comprehensive cohort provided informed written consent.^24^

The present study was approved by the research ethics board of the McGill University Health Centre. We followed the Strengthening the Reporting of Observational Studies in Epidemiology (STROBE) reporting guideline.

### Neuropsychological Assessment

Thorough neuropsychological testing was performed by a trained staff member at baseline and after 3 years.^25,26^ The battery consisted of 10 standard English and French cognitive tests, assessing 3 distinct cognitive domains: memory, executive functions, and psychomotor speed, selected for relevance to diseases of aging and psychometric properties. Memory was assessed using the 15-word Rey auditory-verbal-learning test (RAVLT). Results of the first trial (immediate recall) and second trial (5-minute delayed recall) were used. Executive functions were evaluated using 4 tests: the mental-alternation test (MAT), high interference (color names in incongruent colors/colored dots) of the Stroop test, the animal-fluency test (AFT), and the controlled-oral-word-association test for the letters F, A, and S (COWAT). For the present analysis, the sum of words across the 3 letters was calculated to provide a single total COWAT result. Psychomotor speed was assessed using computer-administered choice reaction times. The mean response time was used for analyses. The RAVLT, MAT, and AFT were administered in home and the COWAT, CRT and Stroop during the interviews at a CLSA data collection site.

### Anthropometry

Anthropometric assessments by trained health assessors included body weight measured wearing light clothing without shoes (140-10 Healthweight Digital Physician Scale; kg), standing height with heels, buttocks, and shoulder blades touching the stadiometer (Seca 213; cm). All were measured to the nearest 0.1 unit and the mean of 2 measurements for weight and height was used.

### Body Composition

Whole body composition, including lean soft tissue mass (lean mass) and fat mass, was measured at baseline using DXA (Hologic Discovery A densitometer) as per standard procedures.^27^ ALM (kg), composed of at least 95% skeletal muscle, was computed as the sum of the upper and lower limbs lean mass and ALM index (ALMI) calculated by dividing ALM by height squared (m^2^). The Canadian cut points for low ALMI^3^ that define sarcopenia were applied: less than 7.30 kg/m^2^ for male individuals, and less than 5.42 kg/m^2^ for female individuals. Body fat percentage was calculated as fat mass (kg) divided by total body weight (kg) multiplied by 100. In the CLSA, DXA contraindications were weight exceeding 204 kg, height exceeding 1.88 m, and exposition to an x-ray with contrast material or participation in a nuclear medicine study within the past 7 days (for accuracy of the measurements). Each DXA body weight was ascertained from weight measured by a scale and only participants with weight agreement were included in analyses.^3^

### Other Covariates

Questionnaires on sociodemographic and lifestyle characteristics were collected at baseline by phone interviews or in person. Demographic variables included sex, language (English or French), level of education (categorical), household income (categorical), and ethnicity (White, other [people from minoritized ethnic groups were combined into a single category because they represented a very low proportion of the total; the other ethnic category included: Arab, Black, Chinese, Filipino, Inuit, Japanese, Korean, Latin American, Métis, North American Indian, South Asian, Southeast Asian, West Asian, and other). Cigarette smoking (current daily, occasional, never) and alcohol use (almost every day, 4-5 times per week, 2-3 times per week, once per week, 2-3 times per month, once per month, less than once per month or never) were self-reported. Social participation was based on the frequency of community-related activities practiced in the last 12 months (none, yearly, monthly, weekly, daily). Symptoms of depression were evaluated with the Center for Epidemiological Studies Short Depression Scale (CES-D10), which is scored from 0 to 30 with higher scores denoting more depressive symptoms^28^; physical activity level using the Physical Activity Scale for Elderly (PASE), a higher score indicating greater level of physical activity^29^; and the risk of poor nutritional state using the abbreviated version of the Seniors in the Community Risk Evaluation for Eating and Nutrition (SCREEN II-AB), which is scored 0 to 48 with lower scores indicating a greater risk.^30^ Presence of type 2 diabetes was self-reported. Whole blood hemoglobin A_1C_ and serum triglycerides were measured.^23^ Grip strength was assessed by handheld dynamometry (kg; Tracker Freedom Wireless Grip). The highest value of 3 trials was used in analyses.

### Statistical Analysis

Characteristics of participants with and without low ALM at baseline were compared by *t* test for normally distributed continuous variables, Mann-Whitney *U* test for nonnormally distributed continuous variables, and χ^2^ tests for categorical variables. Cognitive test results measured in time units were converted to negative (Stroop high interference and choice reaction time) for all scores to have the same orientation (ie, a higher score indicating better cognitive performance). For each cognitive test, the 3-year change was calculated as the difference between year 3 and baseline score, and it was standardized to a *z* score for comparison between tests within a domain. Composite scores of the change in memory (2 results) and executive functions (4 results; COWAT, MAT, animal naming, and Stroop high interference) domains were computed as means. The change in psychomotor speed domain was represented by a single test (choice reaction time). Baseline composite scores per domain were also computed and used as a covariate. Multiple linear regressions were used to examine the association between low ALM and cognitive change in each domain separately. Three models were applied to the 3 cognitive domains. Covariates considered are those available in CLSA and known to affect exposure and/or outcome. Model 1 was adjusted for age, sex, education, language, baseline composite score; model 2 for ethnicity, income, smoking, alcohol use, symptoms of depression, type 2 diabetes, hemoglobin A_1C_ level, serum triglycerides, physical activity, nutritional risk, and body fat percentage in addition to model 1 covariates; model 3 was further adjusted for handgrip strength (continuous). Multicollinearity was verified using Pearson correlations and variation inflation factor (VIF). Results were reported in difference between those with vs without low ALM in pooled nonstandardized mean scores (β) and 95% CI. Two-sided *P* < .05 was accepted as statistically significant.

The proportion of missing data for baseline covariates ranged from 0.3% to 9.3%, and the proportion of missing follow-up cognitive test scores was 16% to 24%. To account for missing information and to reduce bias, multiple imputation with 30 replications was applied. The Markov-chain-Monte-Carlo algorithm was used to impute data; the model included covariates from model 3 and 3-year change in each cognitive test score. Results were reported both as complete case analysis and following multiple imputation, pooled using Rubin rules. The CLSA sample weights were considered in all analyses for results to be representative of the Canadian population. All data analyses were performed using SPSS Statistics version 27 (IBM Corp) from April 24 to August 12, 2020.

## Results

Baseline characteristics of the 8279 participants (4003 female participants [48%]; 8005 White participants [97%]; mean [SD] age: 72.9 [5.6] years) including baseline cognitive test scores are summarized in Table 1; 6681 (81%) were English-speaking and 5979 (72.5%) were highly educated with postsecondary degree or diploma, and the mean (SD) body mass index (BMI, calculated as weight in kilograms divided by height in meters squared) was 27.7 (4.7). A total of 1605 participants (19.4%) had low ALM at baseline. Participants with low ALM were more likely to be men and current daily smokers; they were older, had lower BMI, and lower physical activity level compared with those not having low ALM (Table 1). No differences in education, income levels, and prevalence of type 2 diabetes were observed. At baseline, individuals with low ALM had lower immediate and delayed recall (memory), animal naming score (executive function), and had poorer performance at the choice reaction time (psychomotor speed).

**Table 1.** Baseline Characteristics of Participants in the CLSA Cohort and by Muscularity^a^

Characteristic	Participants, No. (%)			*P* value^b^
	All (N = 8279)	Without low ALM (n = 6674)	With low ALM (n = 1605)	
Sex, No. (%)				
Female	4003 (48)	3393 (84.8)	610 (15.2)	<.001^c^
Male	4276 (52)	3281 (76.7)	995 (23.3))	
Age, mean (SD), y	72.9 (5.6)	72.2 (5.4)	74.3 (5.8)	<.001
Ethnicity				
White	8005 (97)	6476 (97)	1529 (95)	<.001^c^
Other^d^	274 (3)	198 (3)	76 (5)	
French language, No. (%)	1598 (19)	1232 (18.5)	366 (22.8)	<.001^c^
BMI, mean (SD)	27.7 (4.7)	28.6 (4.5)	23.6 (2.9)	<.001
Education				
Less than secondary school graduation	705 (8.5)	567 (8.5)	138 (8.6)	.61^c^
Secondary school graduation, no postsecondary	903 (10.9)	741 (11.1)	162 (10.1)	
Some postsecondary education	692 (8.4)	550 (8.2)	142 (8.8)	
Postsecondary degree or diploma	5979 (72.2)	4816 (72.2)	1163 (72.5)	
Income, $				
<20 000	452 (5.5)	353 (5.3)	99 (6.2)	.17^c^
20 000-50 000	2672 (32.3)	2135 (32.0)	537 (33.5)	
50 000-100 000	3548 (42.9)	2861 (42.9)	687 (42.8)	
100 000-150 000	1078 (13.0)	892 (13.4)	186 (11.6)	
≥150 000	529 (6.4)	433 (6.5)	96 (6.0)	
Type 2 diabetes	918 (11)	759 (11)	159 (10)	.09^c^
Hemoglobin A_1C_, %	5.7 (0.7)	5.8 (0.7)	5.7 (0.6)	<.001
Triglycerides, mmol/L	1.7 (0.9)	1.8 (0.9)	1.6 (0.8)	<.001
Physical activity (PASE)	118.7 (55.9)	120.1 (56.2)	112.8 (54.2)	<.001
Depression scale (CES-D 10; 0-30)	4.7 (4.1)	4.7 (4.0)	4.8 (4.1)	.12
Nutritional risk (SCREEN II; 0-40)	39.2 (5.6)	39.3 (5.6)	38.8 (5.8)	.005
Alcohol consumption, almost everyday	1876 (22.7)	1462 (21.9)	414 (25.8)	<.001^c^
Current daily smoker	423 (5.1)	290 (4.3)	133 (8.3)	<.001^c^
Cognitive test scores				
Memory				
Rey immediate recall, n words (0-15)	5.3 (1.8)	5.4 (1.8)	5.0 (1.7)	<.001
Rey delayed recall, n words (0-15)	3.4 (2.0)	3.5 (1.9)	3.1 (1.9)	<.001
Executive functions				
Animal naming, n words	17.9 (5.2)	18.2 (5.2)	17.6 (5.1)	<.001
MAT (0-51)	24.6 (8.5)	25.1 (8.5)	24.7 (8.0)	.06
COWAT total, n words	37.4 (12.7)	37.8 (12.6)	37.7 (13.1)	.25
Stroop high interference, s	2.32 (0.70)	2.30 (0.67)	2.32 (0.79)	.68
Psychomotor speed				
Choice reaction time, ms	875.2 (193.4)	866.7 (187.9)	884.2 (215.5)	<.001

Abbrevations: ALM, appendicular lean soft tissue mass; BMI, body mass index (calculated as weight in kilograms divided by height in meters squared); CLSA, Canadian Longitudinal Study on Aging; CES-D 10, Center for Epidemiological Studies Short Depression Scale; COWAT, the controlled-oral-word-association test; MAT, Mental Alternation Test; PASE, Physical Activity Scale for Elderly; SCREEN II, Seniors in the Community Risk Evaluation for Eating and Nutrition.

SI conversion factors: To convert hemoglobin A_1c_ % to proportion of total hemoglobin, multiply by 0.01; to convert triglycerides level to milligrams per deciliter, divide by 0.0113.

^a^ Missing data (education, 0.3%; income, 7.8%; hemoglobin A_1C_, 9.3%; triglycerides, 9.0%; physical activity, 6.7%; depression, 2.1%; nutritional risk, 7.1%; alcohol consumption, 2.5%; smoking, 0.7%) are imputed.

^b^ *P* values are from Mann-Whitney *U* test unless otherwise indicated.

^c^ From χ^2^ test.

^d^ Other self-reported ethnicity categories included: Arab, Black, Chinese, Filipino, Métis, Inuit, Japanese, Korean, Latin American, North American Indian, South Asian, Southeast Asian, West Asian, and other.

After 3 years (mean [SD]: 2.9 [0.3] years), the mean memory performance increased in the immediate recall (mean change in score [SD]: 0.4 [1.7]) and delayed recall (mean change in score [SD]: 0.3 [1.8]) (both *P* < .001); within the executive function domain, the animal naming, MAT test and Stroop high interference results deteriorated, whereas the COWAT total score increased; psychomotor speed performance did not change significantly (Table 2). Individuals with low ALM experienced a lesser increase in the mean (SD) immediate recall memory test score (0.3 [1.7] vs 0.4 [1.7]; *P* = .04), a greater mean (SD) decrease in the animal naming (−0.6 [4.0] vs −0.3 [4.1]; *P* = .01), MAT test score (−1.4 [6.0] vs −0.9 [6.1]; *P* = .008), and a mean (SD) decrease in the COWAT score (−0.1 [7.7] vs 0.5 [7.3]; *P* = .04) compared with those not having low ALM (Table 2). Complete case analysis showed similar results but not reaching significance for immediate recall and animal naming tests (eTable 1 in the Supplement).

**Table 2.** Three-Year Change in Individual Cognitive Tests of Participants in the CLSA Cohort and by Muscularity

Cognitive test scores^a^	All	Missing, No.(%)	Without low ALM	With low ALM	*P* value^b^
No. (%)	8279	NA	6674 (80.6)	1605 (19.4)	
Memory, mean (SD), words (0-15)					
Rey immediate recall	0.4 (1.7)	1761 (21.3)	0.4 (1.7)	0.3 (1.7)	.04
Rey delayed recall	0.3 (1.8)	1806 (21.8)	0.3 (1.8)	0.2 (1.8)	.07
Executive functions, mean (SD), words					
Animal naming	−0.4 (4.1)	1688 (20.4)	−0.3 (4.1)	−0.6 (4.0)	.01
MAT, score (0-51)	−1.0 (6.1)	1999 (24.1)	−0.9 (6.1)	−1.4 (6.0)	.008
COWAT total	0.4 (8.1)	1364 (16.5)	0.5 (7.3)	−0.1 (7.7)	.04
Stroop high interference, s^c^	0.03 (0.70)	1333 (16.1)	0.03 (0.70)	0.03 (0.70)	.86
Psychomotor speed					
Choice reaction time, mean (SD), ms	−7.5 (175.9)	1572 (19.0)	−8.4 (172.0)	−3.9 (191.3)	.42

Abbrevations: ALM, appendicular lean soft tissue mass; COWAT, the controlled-oral-word-association test for the letters F, A, and S; MAT, Mental Alternation Test; NA, not applicable.

^a^ Mean (SD) test score values are from multiple imputations.

^b^ *P* values are from *t* test following Rubin rule pooling.

^c^ An increase in the Stroop high interference and choice reaction time indicates a decrease in cognitive performance.

**Figure 2.**
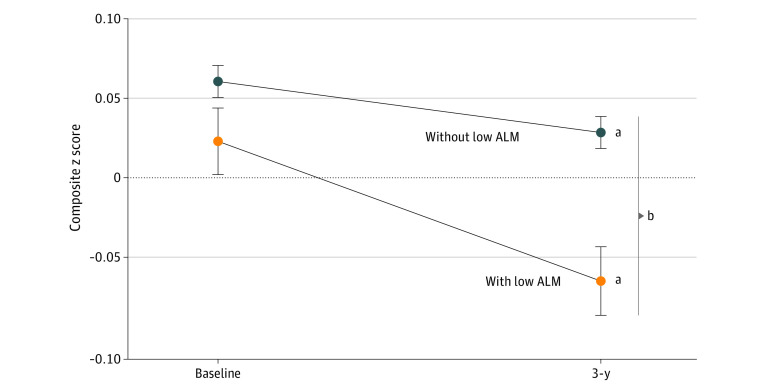
Three-Year Decline in Executive Function, by Muscularity Figure shows mean (SE) from complete case analysis, nonadjusted for covariates. ALM indicates appendicular lean soft tissue mass. ^a^*P* < .001, change within group from paired *t* tests. ^b^*P* < .001, difference in change between groups from independent *t* test.

**Figure 3.**
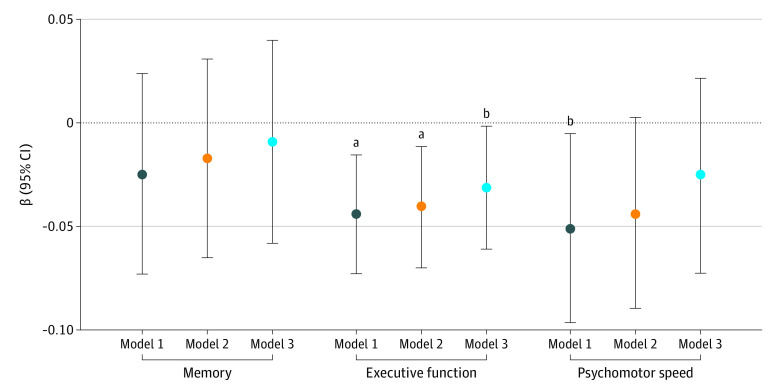
Linear Regressions of the Association Between Low Muscle Mass and Cognitive Decline Over 3 Years by Cognitive Domain Model 1 was adjusted for age, sex, education, language, and baseline cognitive composite score (memory, executive functions or psychomotor speed respectively). *R*^2^ for memory was 0.18; executive function, 0.14; and psychomotor speed, 0.37. Model 2 was adjusted for model 1 covariates and ethnicity, social participation, physical activity, income, alcohol consumption, smoking, blood hemoglobin A_1C_, triglycerides, type 2 diabetes, percentage fat mass. *R*^2^ for memory was 0.18; executive function, 0.14; and psychomotor speed, 0.39. Model 3 was adjusted for model 2 covariates and grip strength. *R*^2^ for memory was 0.18; executive function, 0.15; and psychomotor speed, 0.40. Missing data are from multiple imputation. Analytic weights were considered in all analyses. A negative β is indicative of a greater cognitive score decline in individuals with low muscle mass. ^a^*P* < .01. ^b^*P* < .05.

Figure 2 illustrates the nonadjusted significant 3-year decline in the executive function composite *z* score in individuals with and without low ALM, showing significantly greater decline in participants with ALM. For all 3 cognitive domains, respective baseline composite score was the strongest variable associated with the 3-year change; participants with a lower baseline cognitive score experienced a greater decline. Age (younger associated with lesser decline [standardized β: −0.208; *P* < .001; model 3), sex (men associated with greater decline [standardized β: 0.238; *P* < .001; model 3]) and income (higher associated with lesser decline [standardized β: 0.065; *P* < .001; model 3) were the strongest factors associated with the change in memory performance. Whereas low ALM was not independently associated with memory change (standardized β: −0.010; *P* = .47; model 3) (Figure 3; eTable 2 in the Supplement), grip strength was (β: 0.049; *P* = .03; model 3). Age (β: −0.115; *P* < .001; model 3) and education (higher education associated with lesser decline [standardized β: 0.061; *P* < .001; model 3) were the strongest variables associated with changes in executive function. The presence of low ALM was associated with greater decline in executive functions independently of all covariates including physical activity level, grip strength, and body fat percentage (standardized β: −0.032; *P* = .03; model 3) (Figure 3; eTable 2 in the Supplement). Age (β: −0.077; *P* < .001; model 3) and education (standardized β: 0.048; *P* < .001; model 3) were also the main factors associated with psychomotor speed change. Low ALM was significantly associated with a decline in the latter domain in model 1 (standardized β: −0.025; *P* = .01) (Figure 3), but the association did not remain after full adjustment for covariates (standardized β: 0.007; *P* = .52; model 3) (Figure 3). Similar results were obtained from complete case analysis (eTable 3 in the Supplement). From the 4 individual executive function tests, declines in animal naming (β: −0.36 [95% CI, −0.60 to −0.13]) and MAT were associated with low ALM (β: −0.40 [95% CI −0.76 to −0.04]) whereas changes in COWAT (β: −0.09 [95%CI, −0.58 to 0.40]) and Stroop high interference were not (β: 0.015 [0.06 to −0.03], respectively).

## Discussion

This large longitudinal cohort study with detailed cognitive assessments found that community-dwelling older adults living with low muscle mass (low ALMI) may experience greater cognitive decline, more specifically of the executive function domain, over 3 years compared with persons not having low muscle mass. This association of low muscularity with change in executive function was independent of important related factors including body fat percentage and grip strength. Grip strength, included in the final model, attenuated associations of several covariates, including physical activity that was no longer significant, but low muscle mass remained associated with executive function decline.

To our knowledge, the present study is the first to identify an association between low muscle mass and subsequent cognitive decline, independently of strength typically combined in the sarcopenia construct and thought to drive the association.^17,31^ Indeed, recent evidence from the UK Biobank cohort supports that low handgrip strength estimates increased dementia risk and mortality.^13^ Cross-sectional associations between sarcopenia and cognitive impairment are accumulating^32,33^ and support a positive link between conditions. Two recent meta-analyses found that persons with sarcopenia (heterogeneous definitions) were 2.3 times more likely to have concomitant cognitive impairment compared with those without sarcopenia (adjusted models: 95% CI, 1. 2 to 4.2; n = 5994; 6 studies^32^; and 95% CI, 1.7 to 3.0; n = 10 710; 11 studies^33^). However, very few longitudinal studies are available to date. In a subsample of the EPIDOS cohort, the 7-year change in percentage body fat and muscle mass was not related to new onset cognitive impairment at 7-year follow-up, possibly due to low power (total n = 181 women aged at least 75 years; n = 15 with incident dementia; n = 6 with incident mild cognitive impairment [MCI]).^21^ Another study of limited sample size did not report a significant link between ALM, strength, and MCI or dementia (total n = 297 men and women aged at least 65 years, n = 50 with incident MCI, n = 5 with incident dementia).^34^ Our findings may explain previous reports linking low BMI in older age with greater cognitive decline^35,36^ and incidence of dementia^37^; the low BMI likely resulting from involuntary weight loss that typically includes muscle loss.^38^

Among the 3 cognitive domains assessed, independent associations with sarcopenia were only observed for executive functions and not for memory nor psychomotor function. Executive functions are vital and involved in task initiation, problem solving, attention, organization, working memory, inhibition, and others. Accelerated decline in these functions may interfere with basic and instrumental daily living activities such as financial and shopping skills earlier in life.^39^ Few studies examined associations of sarcopenia with cognitive domains and none in a longitudinal design. One study performed in a Korean population (n = 1887, aged 70 to 84 years) found cross-sectional associations between the Asian Working Group on Sarcopenia (AWGS)-defined sarcopenia (combination of low muscle mass and strength and/or physical performance; odds ratio: [OR], 2.98; 95% CI,1.51-5.89) or the European Working Group on Sarcopenia in Older Person 2 (EWGSOP-2)-sarcopenia (OR, 2.78; 95% CI, 1.45-5.31) and impaired executive functions in men only; however, low muscle mass alone (AWGS criteria) was not associated with any cognitive domains.^31^ The absence of longitudinal investigation prevents direct comparison with our results.

Greater muscle mass may lead to and result from physical activity and cardiorespiratory fitness, hence more blood flow to the brain,^40^ shown to favor executive functioning particularly.^41^ Our statistical models accounted for baseline physical activity level, thus the independent association of low ALM suggests that components specific to skeletal muscle as an endocrine organ may play a protective role in maintaining cognitive executive functions. Indeed, the induction of muscle contraction through exercising may stimulate the release of myokines IL-6 (pleiotropic), IL-8, IL-15, and Brain-Derived Neurotrophic Factor (BDNF) with anti-inflammatory effects^14^ and may partly explain the potential protective effect of preserving muscle mass for brain health. Additional to the hormonal theory, insulin resistance, oxidative stress, and low-grade chronic elevation of pro-inflammatory markers may be involved in both pathogeneses of sarcopenia and dementia.^9,42^ Whether low muscle mass is an early marker or a causal factor of executive cognitive decline, and elucidation of mechanisms linking muscle mass to cognitive functions remain to be determined.

Numerous strengths pertain to this study, lending confidence in the observed results. These include data collected in a large and modern cohort of older adults and sample weights applied in all analyses allowing generalizability to the Canadian population. Also, 7 objective, valid, and reliable (potentially excluding the modified version of the RAVLT) cognitive tests were used, more than in most studies.^26^ Lastly, lean soft tissue mass was evaluated using precise and accurate DXA, the reference method for estimating muscle mass^43^ and study population-specific empirical low ALM cut points were applied.^3^ The current study design is a limitation as it prevents causal inference. However, our results were consistent before and after statistical adjustment for many key confounding factors, rendering alternative explanations for the observed associations less likely plausible. Finally, models were not adjusted for *apolipoprotein E (APOE4)* as it was not measured at the time of our analyses.

### Limitations

This study has some limitations. Surprisingly, mean scores of both immediate and delayed memory tests increased over 3 years in sarcopenic and non-sarcopenic individuals, whereas memory loss is typically expected with aging.^44^ Although the RAVLT test is sensitive in cognitive impairment detection,^45^ our findings may be due to a retest effect or time-saving modifications brought to the RAVLT test in the CLSA (1 trial vs 5 in the original version and 5-minute vs 30-minute delay)^46^ potentially impairing reliability. The increase in the immediate recall was nonetheless significantly blunted in individuals with sarcopenia vs those without, suggesting a deficit in the memory domain. The overall improvement during follow-up in both memory scores may have obscured identification of such deficits and their association with sarcopenia. Also, the number of tests to assess each domain differed with more executive functions tests available, which may have introduced bias. It remains possible that the memory and psychomotor speed domains are affected upon further repeated measures and this can be addressed as future follow-up data become available in CLSA.

## Conclusions

This cohort study found that the presence of low muscle mass measured by DXA was significantly and independently associated with faster subsequent executive function decline over 3 years among adults aged at least 65 years. Importantly, DXA is widely available and measures of lean mass could be routinely incorporated into the image analyses. Clinical screening of older adults to identify those with low muscle mass may provide insight regarding their risk of developing cognitive impairment and thereby guide the testing and application of preventative or therapeutic interventions.

## Notes

Supplement. **eTable 1.** Three-Year Change in Individual Cognitive Tests of Participants in the CLSA Cohort and by Muscularity (Complete Case Analysis) **eTable 2.** Linear Regression Models of the Association Between Low Muscularity and Cognitive Decline Over 3 Years by Cognitive Domain (From Multiple Imputations) **eTable 3.** Linear Regression Models of the Association Between Low Muscularity and Cognitive Decline Over 3 Years by Cognitive Domain (Complete Case Analysis)
