# Supplementary material for: Association of Low Muscle Mass With Cognitive Function During a 3-Year Follow-up Among Adults Aged 65 to 86 Years in the Canadian Longitudinal Study on Aging
Source: JAMA Netw Open. 2022 Jul 1;5(7):e2219926. doi: 10.1001/jamanetworkopen.2022.19926 (PMC9250053; doi:10.1001/jamanetworkopen.2022.19926)
Supplement: Supplement. — eTable 1. Three-Year Change in Individual Cognitive Tests of Participants in the CLSA Cohort and by Muscularity (Complete Case Analysis) eTable 2. Linear Regression Models of the Association Between Low Muscularity and Cognitive Decline Over 3 Years by Cognitive Domain (From Multiple Imputations) eTable 3. Linear Regression Models of the Association Between Low Muscularity and Cognitive Decline Over 3 Years by Cognitive Domain (Complete Case Analysis) [file jamanetwopen-e2219926-s001.pdf]

Supplemental Online Content

Tessier AJ, Wing SS, Rahme E, Morais JA, Chevalier S. Assessment of low muscle mass with cognitive function during a 3-year follow-up among adults aged 65 to 86 years in the Canadian Longitudinal Study on Aging. *JAMA Netw Open.* 2022;5(7):e2219926. doi:10.1001/jamanetworkopen.2022.19926

eTable 1. Three-Year Change in Individual Cognitive Tests of Participants in the CLSA Cohort and by Muscularity (Complete Case Analysis)

eTable 2. Linear Regression Models of the Association Between Low Muscularity and Cognitive Decline Over 3 Years by Cognitive Domain (From Multiple Imputations)

eTable 3. Linear Regression Models of the Association Between Low Muscularity and Cognitive Decline Over 3 Years by Cognitive Domain (Complete Case Analysis)

This supplemental material has been provided by the authors to give readers additional information about their work.

© 2022 Tessier AJ et al. *JAMA Network Open.*

eTable 1. Three-year change in individual cognitive tests of participants in the CLSA cohort and by muscularity (complete case analysis)

Cognitive test scores	All	Without low ALM	With low ALM	P
No. (%)	5331	4339 (81.4)	992 (18.6)	
**Memory**				
Rey immediate recall, n words (0-15)	0.4 (1.9)	0.5 (1.9)	0.3 (1.9)	0.07
Rey delayed recall, n words (0-15)	0.3 (1.9)	0.3 (1.9)	0.2 (1.9)	0.30
**Executive functions**				
Animal naming, n words	-0.3 (4.4)	-0.3 (4.4)	-0.5 (4.3)	0.10
MAT, (0-51)	-1.1 (6.6)	-0.9 (6.6)	-1.8 (6.4)	<0.001
F-A-S total, n words	0.4 (8.0)	0.5 (7.9)	-0.2 (8.2)	0.02
Stroop's high interference, s	0.02 (0.7)	0.02 (0.72)	0.03 (0.75)	0.58
**Psychomotor speed**				
Choice reaction time, ms	-3.2 (179.5)	-5.5 (178.8)	6.6 (182.1)	0.06

Values are Mean (SD). P values are from t-test. An increase in the Stroop's high interference and choice reaction time indicates a decrease in cognitive performance. MAT, Mental Alternation Test.

© 2022 Tessier AJ et al. *JAMA Network Open.*

eTable 2. Linear regression models of the association between low muscularity and cognitive decline over 3 years by cognitive domain (from multiple imputations)

	Model 1		Model 2		Model 3	
	β coefficient (95% CI)	P	β coefficient (95% CI)	P	β coefficient (95% CI)	P
Memory	-0.025 (-0.075, 0.025)	0.31	-0.017 (-0.067, 0.033)	0.49	-0.008 (-0.058, 0.042)	0.73
Executive function	-0.047 (-0.077, -0.017)	0.002	-0.042 (-0.072, -0.012)	0.005	-0.034 (-0.064, -0.004)	0.026
Psychomotor speed	-0.051 (-0.096, -0.006)	0.03	-0.044 (-0.091, 0.003)	0.06	-0.026 (-0.073, 0.021)	0.28

Unstandardized beta coefficients from multiple imputations, as illustrated in Figure 3.

eTable 3. Linear regression models of the association between low muscularity and cognitive decline over 3 years by cognitive domain (complete case analysis)

	Model 1		Model 2		Model 3	
	β coefficient (95% CI)	P	β coefficient (95% CI)	P	β coefficient (95% CI)	P
Memory	-0.023 (-0.072, 0.026)	0.36	-0.037 (-0.096, 0.022)	0.22	-0.024 (-0.083, 0.035)	0.43
Executive function	-0.051 (-0.082, -0.020)	0.002	-0.056 (-0.093, -0.019)	0.003	-0.045 (-0.082, -0.008)	0.022
Psychomotor speed	-0.065 (-0.115, -0.015)	0.01	-0.048 (-0.108, 0.012)	0.11	-0.023 (-0.084, 0.038)	0.45

Unstandardized beta coefficients.

© 2022 Tessier AJ et al. *JAMA Network Open.*
